# Analysis of malaria parasite phenotypes using experimental genetic crosses of *Plasmodium falciparum*

**DOI:** 10.1016/j.ijpara.2012.03.004

**Published:** 2012-05-15

**Authors:** Lisa C. Ranford-Cartwright, Jonathan M. Mwangi

**Affiliations:** Institute of Infection, Immunity and Inflammation, College of Medical, Veterinary and Life Sciences, University of Glasgow, Sir Graeme Davies Building, 120 University Place, Glasgow G12 8TA, Scotland, UK

**Keywords:** Malaria, Plasmodium, Experimental crosses, Quantitative trait loci, Genetic mapping, Recombination

## Abstract

We review the principles of linkage analysis of experimental genetic crosses and their application to *Plasmodium falciparum*. Three experimental genetic crosses have been performed using the human malaria parasite *P. falciparum*. Linkage analysis of the progeny of these crosses has been used to identify parasite genes important in phenotypes such as drug resistance, parasite growth and virulence, and transmission to mosquitoes. The construction and analysis of genetic maps has been used to characterise recombination rates across the parasite genome and to identify hotspots of recombination.

## Graphical abstract

**Graphical abstract:**

**Figure:**
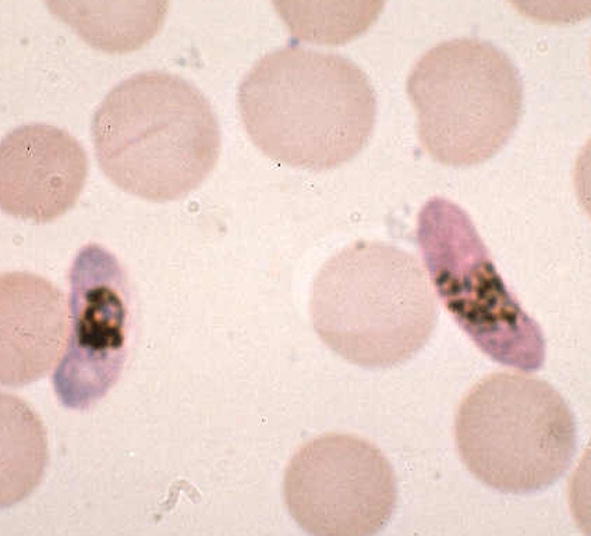

**Highlights:**

► The principles of genetic linkage analysis of experimental genetic crosses in *Plasmodium falciparum*. ► Genetic maps and recombination parameters. ► Quantitative trait locus analysis of parasite phenotypes..

## 1 Introduction; recombination in the laboratory and in nature

Malaria parasites undergo recombination during meiosis, in a similar way to other eukaryotes. The processes of independent segregation of the 14 chromosomes and crossing over between homologous chromosomes both occur during meiosis in the diploid zygote stage of the parasite (Sinden and Hartley, 1985, Walliker et al., 1987), which is formed after fertilisation of the female gamete by the male in the mosquito stomach. The zygote then develops into an oocyst on the mosquito midgut wall, within which there are multiple rounds of mitosis (see Baton and Ranford-Cartwright, 2005 for a review of the parasite stages in the mosquito).

The haploid sporozoites that develop within an oocyst can therefore possess combinations of chromosomes, and genes along those chromosomes, which did not exist in the gametes (known as recombinants). Meiosis produces recombinants only following fertilisation events between genotypically distinct gametes; fertilisation between a male and female gamete from the same parasite genotype (self-fertilisation) would result in a zygote that was totally homozygous at all loci, and chromosomal segregation and crossing over would produce progeny that were identical to the original gametes (with the possible exception of unequal crossing over events).

In the laboratory, cross- and self-fertilisation occur between gametes in the blood meal of a mosquito feeding on an artificial mixture of gametocytes of two different genotypes, at frequencies determined by random associations between gametes, i.e. there does not appear to be a favouring of self- or cross-fertilisation (Ranford-Cartwright et al., 1993). A similar situation of random mating appears to occur in mosquitoes feeding on naturally infected individuals (Anderson et al., 2000); the occurrence of cross-fertilisation requires the presence of genetically distinct gametocytes within the blood of the person bitten by the mosquito, but this appears to be common, at least in high transmission areas (Babiker et al., 1994, Babiker et al., 1999, Paul et al., 1995, Paganotti et al., 2006, Mzilahowa et al., 2007).

Laboratory crossing experiments are easier to analyse than those occurring during natural transmission, because the genotypes of the gametocytes are known and defined, and the maximum number of alleles at any locus is two. The recombinant progeny can be cloned and analysed for their inheritance of the two parental alleles (Walliker et al., 1987). The frequency of recombination events can then be established and the regions of the genome inherited from each parent described for each progeny clone, thus establishing a genetic map.

Three experimental genetic crosses have been performed to date for the human malaria parasite *Plasmodium falciparum* (Walliker et al., 1987, Wellems et al., 1990, Hayton et al., 2008), using five different clones of the parasite (Table 1). The major limitation on performing further experimental genetic crosses is that there is currently no efficient in vitro system for the liver stages of *P. falciparum*; all crosses to date have involved infecting a chimpanzee with sporozoites, and collection of infected erythrocytes for culture from the chimpanzee blood.

**Table 1:** Experimental genetic crosses of *Plasmodium falciparum*.

Cross date	Parent clones (origin)	No. progeny clones (% non-parental)	No. independent recombinant clones available	Reference
1985	3D7 (The Netherlands)^a^	113 (89%)	55	Walliker et al. (1987)
	HB3 (Honduras)^b^			
1990	HB3 (Honduras)	76 (21%)^f^	35	Wellems et al. (1990)
	Dd2 (Laos)^c^			
2008	7G8 (Brazil)^d^	>200 (>86%)	33	Hayton et al., 2008, Sa et al., 2009
	GB4 (Ghana)^e^			

a 3D7 is a clone (Walliker et al., 1987) of isolate NF54 (Delemarre and van der Kaay, 1979; Ponnudurai et al., 1981).

b HB3 is a clone of isolate H1 (Trager et al., 1981, Bhasin and Trager, 1984).

c Dd2 is a clone of the W2-Mef line (Wellems et al., 1988, Oduola et al., 1988a), which was selected from the W2 clone of the Indochina III isolate originally derived from a Laotian patient who failed chloroquine therapy (Campbell et al., 1982, Oduola et al., 1988b).

d 7G8 is a clone of Brazilian isolate IMTM22 (Burkot et al., 1984).

e GB4 is a clone of isolate Ghana III/CDC (Sullivan et al., 2003).

f As reported in Wellems et al. (1990); subsequent cloning produced more recombinant progeny.

## 2 Linkage analysis studies in *P. falciparum*

Analysis of genetic crosses is an especially useful technique to identify genomic loci influencing parasite phenotypes for which there are no obvious candidate genes, for example, drug resistance where the mechanism of action of the drug is unknown. A major advantage of the analysis of crosses over population association studies is that there are only two alleles (maximum) within the progeny clones in the former – those existing in the two parents used for the cross – whereas there could be very many within a population sample in the latter studies. Therefore, loci can be identified by analysing smaller numbers of parasite clones from an experimental cross, and there are less likely to be confounding effects from multiple forms of multiple genes.

If variation in a parasite phenotype is controlled by polymorphisms within a single gene, each haploid progeny clone will inherit the allele from either one parent or the other, and the progeny will all exhibit one of two variants of the phenotype – two parental-like groups – with no intermediate phenotypes. Any variation in phenotype will be due to environmental differences or to experimental noise. The gene controlling the phenotypic variation of interest can be identified by examining the genomes of the progeny clones, and finding the region(s) of the genome that are shared among the parent and progeny that exhibit one phenotypic state. This is the principle of linkage analysis.

In cases where multiple genes contribute to the phenotypic variation, non-parental phenotypes would be observed, which could fall between the two parental phenotypes, or be greater or smaller than either parent. These multigenic or complex traits are analysed using a statistical approach known as quantitative trait locus (QTL) analysis (Gelderman, 1975).

A linkage analysis study of an experimental genetic cross involves three steps: (i) the phenotype of interest is examined in the progeny of a genetic cross; (ii) a genetic map of each of the progeny clones is generated, based on the inheritance of polymorphic markers, to identify the regions of each chromosome inherited from each of the two parents in the cross; and (iii) the phenotype and genotype data are linked using statistical methods such as maximum likelihood (ML) analysis and Log of Odds (LOD) scores (Fisher, 1922a, Fisher, 1922b, Haldane and Smith, 1947, Barnard, 1949).

### 2.1 Constructing a genetic map

To perform any linkage analysis, it is necessary to identify polymorphic markers throughout the genome and “map” their positions and relative genetic distances along each chromosome (Sturtevant, 1913). The polymorphic markers are usually based on differences in the DNA such as substitutions (single nucleotide polymorphisms or SNPs), rearrangements such as insertions or deletions, or errors in replication of repetitive regions of DNA (e.g. microsatellites) (Botstein et al., 1980, Spierer et al., 1983, Weber and May, 1989, Kruglyak, 1997, Collard et al., 2005). More rapid typing of multiple markers within a parasite clone can be achieved using high throughput methods such as microarrays (Jiang et al., 2008).

Initial analysis of experimental crosses of *P. falciparum* used genetic maps based on restriction fragment length polymorphism (RFLP) markers (Walker-Jonah et al., 1992). Denser maps were constructed using hundreds of polymorphic microsatellite markers (Su and Wellems, 1996, Su et al., 1999), and the most recent maps have utilised thousands of SNPs (Jiang et al., 2011), on platforms such as Affymetrix Molecular Inversion probes and chips (Mu et al., 2010) and Nimblegen (Tan et al., 2010). The density of markers within a map must, however, be carefully considered: more markers do not necessarily improve a map. The algorithms used in linkage analysis assume that alleles at each marker are independent of one another; densely distributed markers (such as SNPs) are more likely to be in linkage disequilibrium, where adjacent SNPs are associated with one another and are not inherited independently. This problem can be overcome using one of several methods proposed to select SNP markers that are in linkage equilibrium (Abecasis et al., 2002, Allen-Brady et al., 2007, Purcell et al., 2007, Thomas, 2007).

### 2.2 Linking phenotype and genotype

**Fig. 1:**
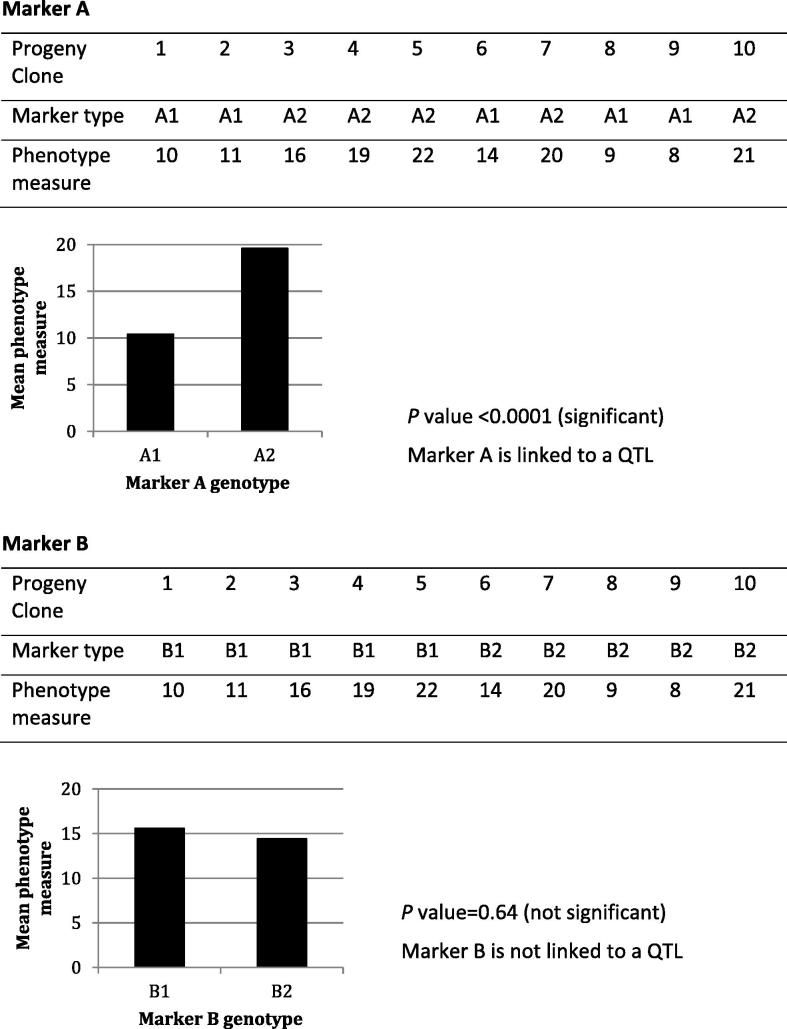
The principle of linkage analysis. Examples of two markers, A and B, and their linkage to a phenotype. The marker type gives the allelic variant of each marker in the progeny clones. Ten progeny clones are shown to illustrate the principles. QTL, quantitative trait locus analysis.

Linkage analysis examines the co-segregation of a chromosomal region and a trait of interest. Basically, for each marker in the map, the progeny clones are sorted into those with one parental allele or the other and the phenotype difference between the two groups is examined (Fig. 1). A significant difference in phenotype between the two marker groups indicates the marker locus being used is linked (near to) the gene controlling the trait. A marker that is not linked to the gene controlling the trait will be randomly inherited with respect to the trait gene, and there will be no significant difference between the mean phenotype of the two marker groups.

Linkage between markers and the trait is usually expressed as a LOD score (log of odds), which represents the likelihood of linkage versus no linkage (Morton, 1955). A LOD score of 3 indicates that linkage is 1,000 times more likely than no linkage i.e. odds of linkage of 1,000 to 1. A LOD score is calculated for each of the markers on the genetic map and LOD scores can also be calculated for two or more markers interacting either in an additive fashion or epistatically. There are several statistical packages to analyse linkage both of simple traits (single locus) or more complex traits involving multiple contributing loci (QTL) (Lander and Botstein, 1989, Lynch and Walsh, 1997, Zou and Zeng, 2008).

Plasmodium crosses have been analysed for single-locus as well as QTL, which will be discussed in Section 4. Analysis is made simpler because the parasite stages undergoing phenotyping are haploid; for a single-locus study there are no heterozygotes and parasite clones are treated as being fully homozygous at all loci. Analysis of experimental genetic crosses has allowed the identification of parasite loci controlling both simple and complex traits, including resistance to antimalarial drugs such as chloroquine and quinine (Wellems et al., 1991, Ferdig et al., 2004), the ability to invade erythrocytes of different primate species (Hayton et al., 2008), and the ability to infect mosquitoes (Mwangi and Ranford-Cartwright, unpublished data).

## 3 Recombination rates and hotspots of recombination

Genetic maps define the relative order of loci along a chromosome in terms of genetic distance, which measures the likelihood of crossover events between those two loci. Crossover events are more likely to occur between markers further apart on a chromosome than between those close together. Crossover events are initiated by the formation of a double strand break (Keeney et al., 1997), which is then repaired using the homologous chromosome as a template. While the genetic map is usually similar to the physical map (where sequence data define the precise position of the loci on the chromosome), there can be differences; for example the genetic distance between two loci will appear greater than the physical distance if there is high recombination activity (a “hotspot”).

The genome-wide rate of recombination varies widely between different organisms, but seems to be high in apicomplexan parasites compared with other eukaryotes e.g. 0.8 Mb/cM for humans, 1.8 Mb/cM for rat (Jensen-Seaman et al., 2004), 4.5 kb/cM for *Theileria parva* (Katzer et al., 2011), 10–56 kb/cM for *Cryptosporidium parvum* (Tanriverdi et al., 2007). The most recent SNP-based genetic map for *P. falciparum* used over 3,000 SNPs (Jiang et al., 2011) to genotype 32 progeny clones from an experimental cross between parasite clones GB4 and 7G8 (Hayton et al., 2008). The map generated allowed an estimate of recombination rate, with a map unit size of 9.6 kb/cM (including the highly recombinogenic subtelomeric regions) or 12.8 kb/cM (excluding the subtelomeric regions) (Jiang et al., 2011), smaller than the previous estimate of 36 kb/cM obtained using 285 microsatellite markers (Hayton et al., 2008). Estimates from other *P. falciparum* genetic crosses are similar: 12.1 kb/cM for the HB3 × Dd2 cross (Su et al., 1999) and ∼11 kb/cM for the 3D7 × HB3 cross (Ranford-Cartwright and Mwangi, unpublished data).

Crossover/gene conversion events are not randomly distributed throughout the genome. In human and yeast genomes, “hotspots” have been identified, which are highly localised short regions (70–250 bp) with relatively high frequencies of crossover and gene conversion events (Gerton et al., 2000, Myers et al., 2005). In contrast to bacterial recombination, which often occurs at a “chi” sequence (Cross-over Hot-spot Instigator) (Smith et al., 1995), there appears to be no consensus sequence for eukaryotic hotspots of recombination in humans. In *P. falciparum*, crossover/gene conversion events are more common in the subtelomeric regions of the chromosome (Mu et al., 2005, Jiang et al., 2011), possibly due to the presence of multi-copy gene families such as *var*, *stevor* and *rifin*. However, recombination during mitosis may contribute to the high frequencies seen for the telomeric *var* (Freitas-Junior et al., 2000). Analysis of non-subtelomeric hotspots identified a 12 bp GC-rich motif with a 3 bp G periodicity (Jiang et al., 2011), which is similar to a motif found in human recombination hotspots that may interact with zinc-finger DNA-binding proteins (McVean, 2010).

The ability to identify candidate genes is dependent on the location of the informative recombination events and an adequate number of markers to capture these recombination events to define the locus. Therefore, the relatively high recombination frequency of *P. falciparum* reduces the number of progeny clones that need to be phenotyped and genotyped to identify QTL to an experimentally manageable number. For example, the *Pfmdv1* locus underlying a male gametocyte defect was initially mapped using just 11 progeny clones (Vaidya et al., 1995).

## 4 Identification of parasite loci through linkage analysis

### 4.1 Genes contributing to drug resistance

Many linkage analysis studies in *P. falciparum* have been performed to identify parasite genes that contribute to drug resistance, starting with chloroquine resistance. The *Pfcrt* gene was mapped initially, using 16 progeny clones from the cross between the chloroquine-sensitive parasite HB3 and the chloroquine-resistant clone Dd2, as a 400 kb locus on chromosome 7 (Wellems et al., 1991). Isolation and analysis of a further 19 progeny clones narrowed the locus to a 36 kb segment containing nine open reading frames (ORFs) (Su et al., 1997). A highly fragmented gene, denoted *Pfcrt*, was subsequently discovered within the 36 kb locus; a specific mutation causing a lysine to threonine change at position 76 (K76T) was found to be required for resistance (Fidock et al., 2000, Sidhu et al., 2002). Analysis of a cross between two chloroquine-resistant parasites, GB4 and 7G8 (Hayton et al., 2008), revealed that an additional gene, *Pfmdr1*, contributed to the level of chloroquine resistance (Sa et al., 2009). Further analysis of the HB3 × Dd2 cross showed that *Pfmdr1* also influenced the degree of resistance in chloroquine-resistant progeny of the Dd2 × HB3 cross (Patel et al., 2010).

Analysis of low level quinine resistance in the HB3 × Dd2 cross using QTL techniques revealed contributions from loci on six separate chromosomes (Ferdig et al., 2004). Loci on chromosomes 5, 7 and 11 were found to interact in an additive fashion; these loci contain the *Pfcrt*, *Pfmdr* and the sodium-proton exchanger *Pfnhe* genes, respectively. Further studies confirmed that changes in the level of *Pfnhe* expression affected the response to quinine and that this effect was strain-dependent, which suggested the involvement of other polymorphic genetic loci making up different genetic backgrounds (Nkrumah et al., 2009). There were additional interactive effects between two loci on chromosomes 9 and 6 and the two QTL on chromosomes 13 and 7 (Ferdig et al., 2004), which have not yet been investigated further.

More recently, a high throughput approach was used to screen for growth inhibitors in a library of over 1,000 bioactive chemicals (Yuan et al., 2009). Three chemicals demonstrating differential activity against parasite clones GB4 and 7G8 were analysed further using the progeny clones from the cross between these two clones to identify potential target genes. Parasite sensitivity to trimethoprim (a DHFR inhibitor used to treat urinary tract infections) and triamterene (a Na^+^ channel blocker) mapped to *pfdhfr*, while parasite response to dihydroergotamine methanesulfonate (DHMS, a serotonin receptor antagonist), mapped to *pfmdr1* (Yuan et al., 2009).

### 4.2 Genes contributing to intraerythrocytic growth and invasion

The genes contributing to the ability of *P. falciparum* to invade erythrocytes of the squirrel monkey, *Aotus nancymaae*, were mapped using a QTL approach with the progeny of the GB4 × 7G8 cross (Hayton et al., 2008). The GB4 parasite is highly virulent in *A. nancymaae* monkeys, whereas the 7G8 clone is unable to invade *A. nancymaae* erythrocytes. The invasion trait mapped to a 14 kb region on chromosome 4 containing two genes, *Pfrh4* and *Pfrh5*, and allelic exchange showed that a single polymorphism in the *Pfrh5* gene, which resulted in an amino acid change from isoleucine to lysine at codon 204, conferred the ability to invade *A. nancymaae* erythrocytes.

Parasite growth rates within the erythrocytes also appear to be under genetic control. Analysis of the HB3 × Dd2 cross revealed that 75% of the variation seen in the time taken for a parasite to complete the intraerythrocytic cycle was explained by a major QTL on chromosome 4, two additive loci on chromosome 14, and with additional contributions from two loci on chromosome 4 and 13 that acted epistatically (Reilly et al., 2007, Reilly Ayala et al., 2010).

### 4.3 Genes contributing to parasite transmission

The parasite Dd2 infects mosquitoes poorly, despite producing morphologically normal gametocytes; analysis of 11 progeny from the HB3 × Dd2 cross indicated that this failure to infect mosquitoes was linked to an 800 kb locus on chromosome 12 (Vaidya et al., 1995). Further mapping using an additional eight progeny clones narrowed the locus to an 82 kb region containing 29 predicted genes (Furuya et al., 2005). Comparison of gene expression between the defective Dd2 clone and its non-defective W2 parent clone revealed down-regulation of one gene, denoted *pfmdv-1* (*P. falciparum* male gametocyte development gene 1). Disruption of the *pvmdv-1* gene resulted in a reduction of mature gametocytes and developmental arrest at stage I. Although this was not the exact phenotype seen in the cross, the study identified *Pfmdv-1* as a key contributor to gametocyte development.

QTL analysis has also been carried out to investigate parasite genes contributing to parasite development within the mosquito (Ranford-Cartwright and Mwangi, unpublished data). QTL analysis of progeny from the 3D7 × HB3 cross revealed a major locus on chromosome 12 which accounted for 94% of the variation in infection intensity (number of oocysts per mosquito midgut) and 44% of the variation in infection prevalence (proportion of mosquitoes infected).

### 4.4 Genetic loci affecting gene expression

Although linkage analysis has primarily been used to identify polymorphisms within protein-coding genes that affect phenotype, transcriptional variation can also be measured and mapped using genetic crosses, thus identifying expression QTL (eQTL). Analysis of a *P. falciparum* genetic cross revealed that expression levels of many transcripts during the asexual parasite lifecycle were heritable and could be mapped (Gonzales et al., 2008). Approximately 18% of genes expressed in late ring stage/early trophozoites were regulated by a significant eQTL and both cis- and trans-regulatory loci were identified, including a regulatory hotspot associated with a copy number variant on chromosome 5 that affected expression levels of 269 transcripts within the genome. eQTL analyses therefore can provide additional information on variation in regulatory loci including promoter or transcription factors, but also epigenetic components, copy number variation, non-coding RNAs or signalling cascade variations that are capable of changing transcript pools.

## 5 Conclusions

Analysis of experimental crosses (linkage analysis) remains an important tool in malaria research, allowing unbiased identification of gene(s) affecting a phenotype, rather than a candidate gene approach. Modern genotyping methods and computing power allow more rapid identification of genetic regions controlling a trait and thus have significantly strengthened the power of linkage mapping approaches in recent years. The approach allows the identification of multiple genes contributing to a phenotype, including loci interacting epistatically and additively, and it is possible to estimate the relative contribution of a genetic locus to a phenotype.

Analysis of experimental genetic crosses of *P. falciparum* has allowed the discovery of genetic mechanisms behind a variety of phenotypic traits for which there were no obvious candidate genes. Experimental crosses have advantages over studies using field isolates with diverse genetic backgrounds, for example (i) the limited genetic variation in the progeny (derived from only two parents) avoids possible confounding effects of multiple forms of multiple genes, and (ii) the ability to phenotype laboratory clones repeatedly under controlled conditions can reduce environmental variation (biological and measurement noise).

Progeny clones from the three experimental crosses performed to date, coupled with the high density genetic maps now available for each cross, provide a useful community resource for future work to understand the influence of parasite genetic polymorphism on biological phenotypic variation. Further refinement of the genetic linkage maps, including complete sequence information for the progeny clones, will reveal new crossovers, assist in identifying precise points of recombination (chromosome breakpoints) and hot spots of recombination, and will facilitate positional mapping of candidate genes.
